# Influence of forest stand characteristics on physical, mechanical properties and chemistry of chestnut wood

**DOI:** 10.1038/s41598-020-80558-w

**Published:** 2021-01-15

**Authors:** Francesco Marini, Maria Chiara Manetti, Piermaria Corona, Luigi Portoghesi, Vittorio Vinciguerra, Swati Tamantini, Elena Kuzminsky, Florian Zikeli, Manuela Romagnoli

**Affiliations:** 1 Università degli Studi della Tuscia (DIBAF), Viterbo, Italy; 2 CREA (Consiglio per la ricerca in agricoltura e l’analisi dell’economia agraria), Research Centre for Forestry and Wood, Arezzo, Italy; 3 Institute of Chemical, Environmental and Bioscience Engineering, Technische Universität Wien (AT), Vienna, Austria

**Keywords:** Plant sciences, Environmental sciences, Chemistry, Materials science

## Abstract

Site conditions and forest management affect dendrometric parameters of chestnut (*Castanea sativa* Mill.) coppices, but there is modest knowledge on the effect of stand dendrometric characters on physical and mechanical wood characteristics. The aim of this study was to verify these relationships in chestnut coppices that were 12–14 years old. Wood density, compression and bending strength, shrinkages were measured on shoots of five different stand in a vulcanic site in Monte Amiata (Central – Italy). Investigated stands differ in number of stools/ha and dominant height, diameter/basal area of the shoots. The main difference in the physical characters among the stands is density. The initial results of the study showed that physical, mechanical wood characters are more dependent by the shoot than by the site. There is a positive relationships between the number of stools/ha and density and a negative one among shoot dominant height and basal area with wood density. Spectroscopic profile by FTIR has not showed relevant differences among the stands. Wood anatomy has showed the breakpoint at cellular level.

## Introduction

Wood quality can be described by many characteristics, and according to the classical meaning, it is related to the final use of wood^1–3^, affected by tree phenotype, like stem shape and bearing, stem curvature crown shape development as well as number and size of branches. From a technological point of view, wood quality is related to wood defects but also to some physical and mechanical parameters, like wood density, shrinkage, and mechanical properties^4–6^. In recent times, wood quality based on physical and mechanical properties has also assumed a new meaning because they are considered proxy descriptors of tree resilience to climatic changes^7,8^. The study of the environmental and site characteristics that determine wood physical and mechanical properties is quite complex because of the influence of many other factors, i.e., tree age, genetics, etc.^9,10^, and the inter-correlation among the parameters, such as the most known effects of wood density on mechanical strength^11,12^. Nevertheless, forest management and site descriptors have proved to impact the most representative physical and mechanical features in wood. The research in this field is much more developed in conifers^13–18^ than in hardwood species; the related papers on hardwoods consider forest stands both with ring porous species, like oaks^6,19^ and diffuse porous rings^20^. The few references on coppice and chestnut deal with the effect of silviculture to phenotypic characters and wood defects^21–23^ though certain papers have characterized wood provenance from the chemical point of view^24^ or explored the effect of mechanical properties on ring shake^25^.

In the future, stand attributes derived from silviculture operations will play a major role because of changing wood quality, and they could mitigate the impact of climatic changes, also affecting the wood-based economy of territories. From this point of view, wood chemical characteristics, both considering wood extractives and cell wall main components, are sometimes under-evaluated compared to the more macroscopic characters (physical and mechanical properties) and will assume a crucial role.

Wood chemistry is responsible for many properties, the most important being durability^4,26,27^, which in chestnut seems less connected to the character of wood structure^26^.

Indeed, wood chemistry is quite related to climatic changes and the lignification process in chestnut, which has demonstrated to be site- and climate-dependent^28,29^, meaning the relationships among climate-stand character, wood chemistry and wood properties have a different interconnection compared to the actual state of the art.

The aims of this paper were to validate the influence of site growth conditions as assessed by forest and dendrometric measurements on physical and mechanical wood properties and chemical composition in chestnut coppices. Chemical analysis has been carried out by means of FTIR spectroscopy, which has the major advantage of being quite a speed system to investigate wood chemistry. FTIR spectroscopy associated with wood density measurements has proven to be a promising alternative to traditional methods for screening of individual- or species-specific resistance to embolism in angiosperms^30^. This investigation represents the first attempt at dealing with these aspects for new perspectives on chestnut.

The material of the investigation is wood from young thinned shoots and one of the further possible impacts of the research is to lead to a new perspective on the alternative use of young shoots to firewood and poles with different various final products and the chance to modify the silvicultural approach in chestnut coppice stands^31^ also looking towards a more significant development of the short supply chain^32,33^.

## Results

### Coppice forest characteristics

**Figure 1:**
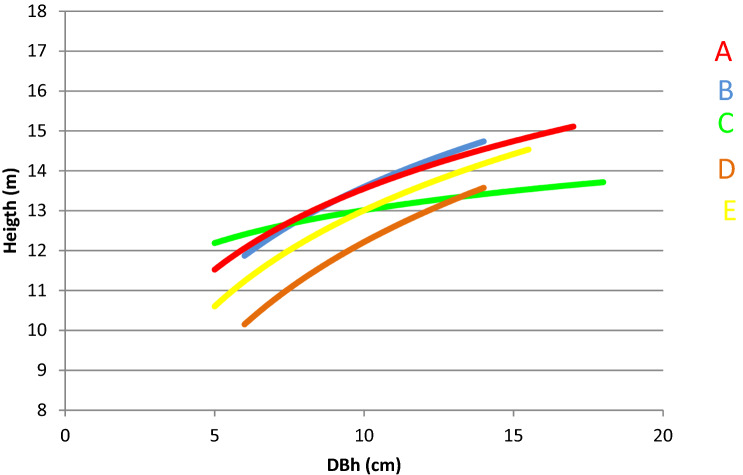
Height-diameter curves of the five chestnut coppices on Amiata mountain. DBH = diameter at breast height.

In Table 1, the main dendrometric attributes of the five sampled coppice stands are shown. The stands, overall, are quite different from each other. Based on field measurements, the relationship among DBH (Diameter et Breast Height) and tree height were performed (Fig. 1). Plots A and B have a similar density in terms of number of stools and shoots, which are significantly lower than the values of plots C and E. Plot D has a number of shoots/ha much lower than the other owing to thinning, which was performed at year 2014 at a reduction of at least 50%. Therefore, it can be thought that the number of individuals before thinning in area D was similar to that of area E.

**Table 1:** Dendrometric parameters of chestnut studied stands on Amiata mountain (in^32^ modified).

Area	C	A	B	D	E
Altitude (m)	1145	990	1030	1100	1030
Exposure	SE	SE	SE	SE	SO
Slope (%)	0	0	30	30	40
Stools/ha	780	580	623	1047	962
Shoots/ha	4810	3480	3860	2845	5210
Shoots Dominant heigth (cm)	13.6	14.9	15.3	13.8	14
Shoots dg (cm)	8.9	9.6	10.6	10	7
Volume m^3^/ha	220	193	269	156	136
G (m^2^/ha)	29.95	25.41	34.2	22.42	20.31

Comparing each other the values of Table 1 and comparing them with the graphs in Fig. 1, it can be assessed that stand density differences are reflected in the average diameter. The lower stand competition allows the shoots to grow larger in diameter.

Plots A and B also had similar values of dominant height, hence site fertility, compared to the other three stands. This is confirmed by the comparison between the height-diameter curves (Fig. 1). In plot C, the differentiation into social classes was less marked, so the curve is flatter, probably because of the high density and low fertility.

With reference to the auxological model of the Cimini Mountains, a group of volcanic reliefs located just south of Mount Amiata in a similar environmental context^34^, a site index slightly higher (A and B sample plots) and slightly lower (C, D and E) than 17 at a reference age of 25 years can be attributed. Therefore, the studied stands grow in conditions of average soil fertility in the context of volcanic reliefs of central-western Italy.

### Physical and mechanical wood properties

The mean values of the physical and mechanical properties of the wood from each site are reported in Table 2.

**Table 2:** Physical and mechanical characteristics of chestnut wood of Amiata provenance.

Area		βV (%)	ρ_12_ (kg/m^3^)	CS (MPa)	MOR (MPa)	HB (N/mm^2^)	Ring widths (cm)
C	Mean	9.98	592	52	88	24	0.44
	SD	1.19	42.70	4.17	14.80	5.24	0.09
A	Mean	10.00	563	49	87	26	0.46
	SD	0.96	38.20	5.13	15.20	2.71	0.11
B	Mean	9.92	557	47	88	22	0.41
	SD	0.78	42.50	3.30	17.70	5.74	0.1
D	Mean	10.68	641	54	98	29	0.41
	SD	1.35	45.70	6.01	18.20	2.19	0.1
E	Mean	10.36	614	51	71	32	0.44
	SD	1.27	34.80	5.49	13.40		0.11

Wood density at 12% moisture (ρ12), total volumetric shrinkage (βV), compression strength (σ12), bending strength (MOR), Brinell hardness (HB) and ring width (RW).

Wood density was highest in the D stand, while the lowest density value was found in B. The D stand also had the highest βV, MOR and σ_12_, while the lowest values were in B (except for MOR). The D site had the highest variability in all the measured physical and mechanical parameters as assessed by standard deviation values (SD).

The ANOVA (Mann–Whitney non-parametric test) reported in Table 3 indicates that the A plot is very similar to B and E both for physical and mechanical wood properties, an high similarity was also assessed in the main dendrometric parameters in^32^. The site that differs most from all others is D, especially for physical and mechanical wood parameters. Namely wood density ρ_12_ is the more frequently different value among the stands. According to Table 3, it can be supposed that diameter and basal area could affect density and compression strength in B and E. Also according to Table 3, it is evident that the number of shoots per stool is significantly different in D, and this is the effect of the thinning. At any rate, in this last case, the different number of shoots in each stool is not related to the differences in the values of wood technological parameters because the measured samples were located in the pre-thinning period (at ages 4–9 approximatively).

**Table 3:** ANOVA of five different chestnut stands on the Amiata mountain, related to dendrometric parameters and physical wood properties.

A	B	C	D	E
A	ρ_12_** CS***	ρ_12_**	SS*** ρ_12_** σ_12_**	D*** BA***
	B		SS*** ρ_12_*** σ_12_*** HB***	D*** BA*** ρ_12_*** σ_12_***
		C	SS*** ρ_12_***	D*** BA***
			D	SS*** ρ_12_** MOR**
				E

Wood density at 12% moisture (ρ12), total shrinkage (βV), compression strength (σ12), bending strength (MOR), Brinell hardness (HB) and ring width (RW).

*D* diameter at breast heigth, *BA* individual basal area, *SS* shoot per stools.

***p < 0,001 **p < 0,01 *p < 0,05.

**Figure 2:**
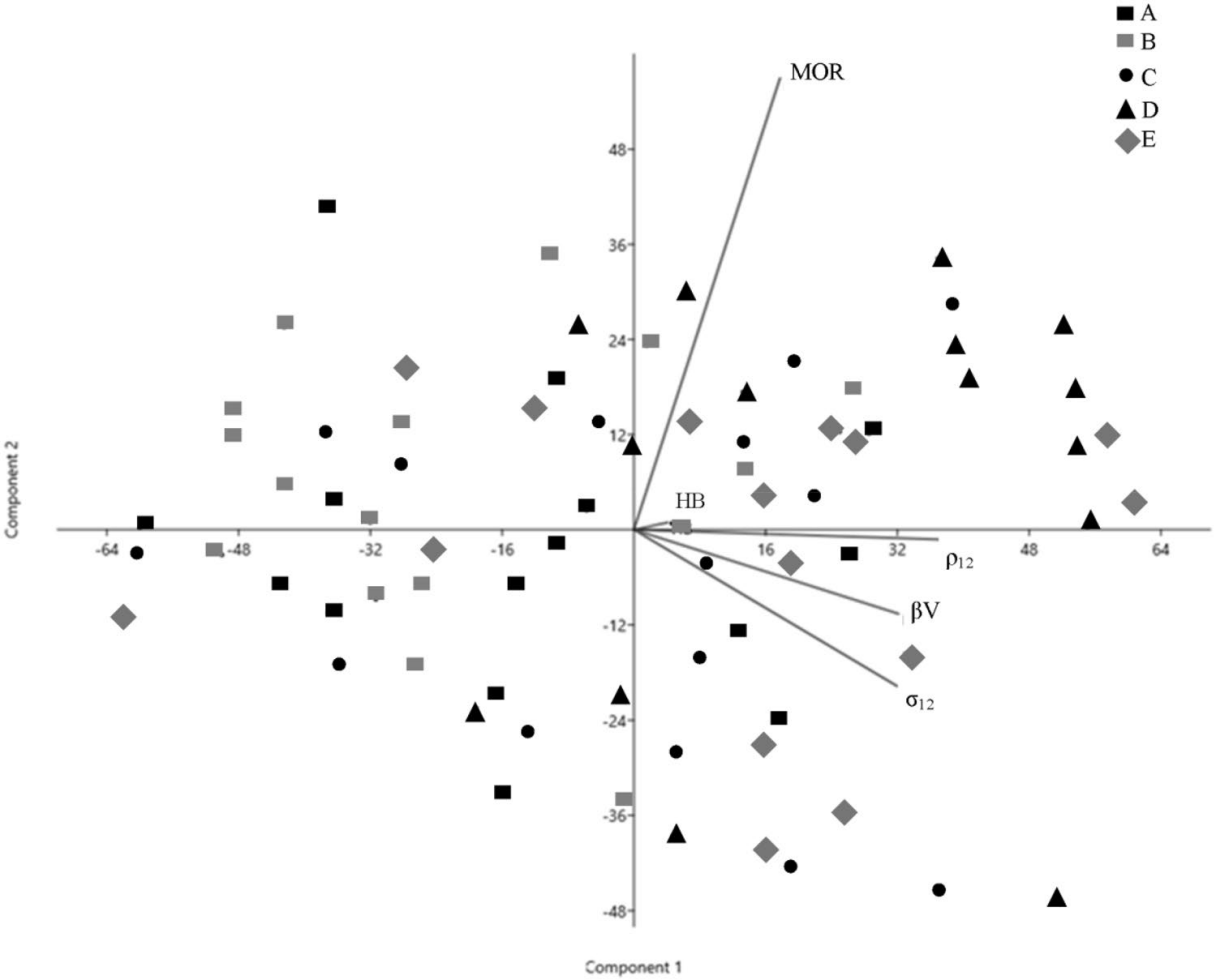
Principal Component Analysis of physical and mechanical wood properties of the five chestnut coppices on Amiata mountain. Wood density at 12% moisture (ρ12), total shrinkage (βV), compression strength (σ12), bending strength (MOR), Brinell hardness (HB).

The PCA of wood physical and mechanical properties does not show there to be a cluster of the samples according to the sites, demonstrating the main differences in MOR on one hand and ρ_12_, σ_12_ and HB on the other are more drived by the individual character of each sample/shoot (Fig. 2).

As the ρ_12_, βV and σ_12_ arrays are in the same direction, they could yield information of a direct strong relationship among the variables, with reasonable shrinkages and compression strength positively affected by wood density.

**Figure 3:**
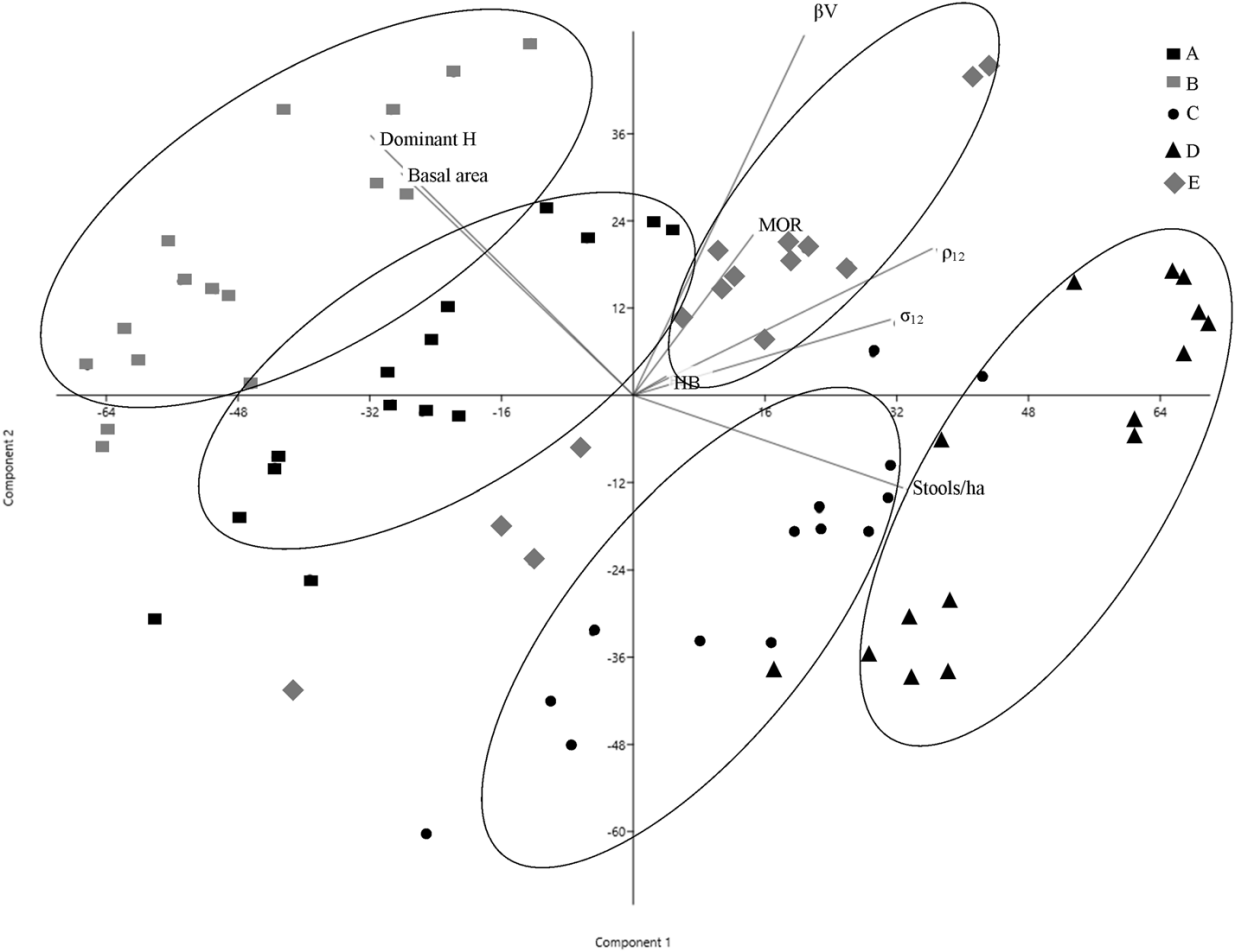
PCA of physical and mechanical wood properties, with principal dendrometric parameters of the five chestnut coppices on Amiata mountain. Wood density at 12% moisture (ρ12), total volumetric shrinkage (βV), compression strength (σ12), bending strength (MOR), Brinell hardness (HB), Individual shoots basal area (Basal area), stools per hectar mean value (Stools/ha), Shoots top height mean value per stand (Dominant H).

Adding to the PCA analysis the dendrometric parameters, such as stools per hectare, dominant height and basal area, it drives a different distribution of the samples that appear a little more grouped together when they belong to the same stand (Fig. 3). By Fig. 3 it can be assessed as there is a lower number of stools per hectare, there is higher growth of the individual shoots expressed in terms of basal area and dominant height and, interestingly, all investigated physical and mechanical properties are lower. This result can be easily checked compared the values in Table 1 because the stands A and B, which have the highest values of basal area and dominant height and a low number of stools per hectare are characterized by low ρ_12_ and the other related physical properties (shrinkages, compression and bending strength).

Pearson correlation between stand physical–mechanical properties with dendrometric parameters supply additional relevant information. ρ_12_ is positively correlated, significantly, to stools per hectare (R = 0.61; *p* value < 0.005) and negatively and significantly correlated to dominant height of shoots (R = − 0.50; *p* < 0.01). Wood density was positively and significantly correlated to the other physical parameters, βV, σ12 and HB, exhibiting correlation coefficients, respectively, of 0.56***, 0.53** and 0.72**.

### Microscopic analysis

**Figure 4:**
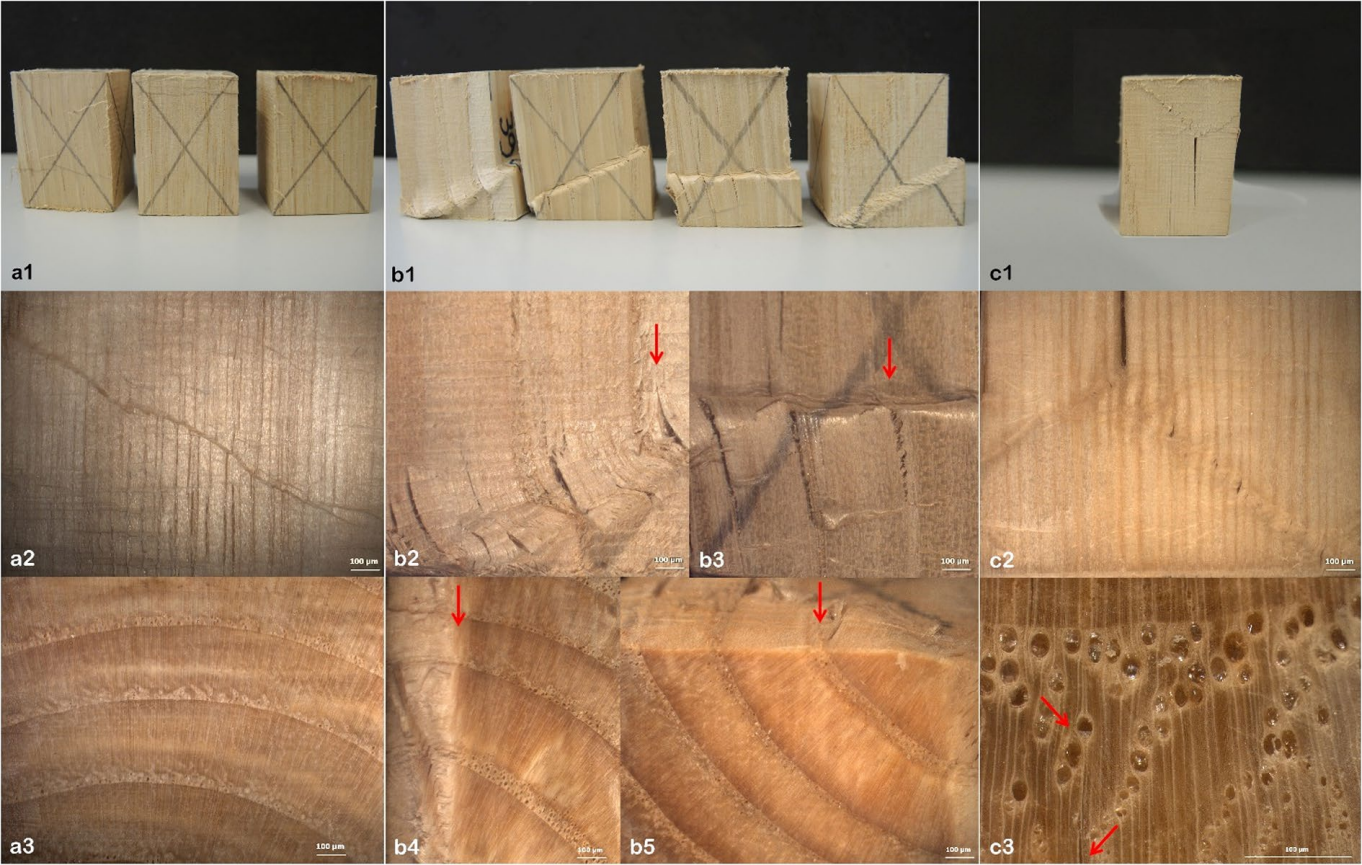
The column (**a**) is with samples showing highest mechanical strength. The break line is not so evident on longitudinal side. In the column (**b**), the typical rupture at compression strength of the most samples. In (**b2**) and (**b3**) it is evident a split in the tissues. In (**b4**) there is an overlapping of radial size in the cross-cut side due to compression stress. In (**b5**) the rupture of (**b3**) is located at the end of ring width in the transition from latewood to the earlywood of the next ring. The rupture in (**c1**) is in the less resistant specimen, the cleavage break occurs in the cell walls of latewood vessels and it is in specimen with more gradual transition from the ring porous to latewood vessels.

In Fig. 4 the images of the break line in the samples are reported. The type of rupture in the tissues is in agreement with the classic shapes reported in^35,36^. The first group (a) represents the samples with highest mechanical compression strength. To the group (b) belong the most samples with the average values of strength in axial compression. The third group (c) is related to the sample with the lowest mechanical strength and it shows an axial split. In the samples with highest strength (a) the break line on longitudinal sections is very thin and no evident deviation or fractures in the tissue is observed. In the samples b, there is a variability in the fractures of the samples, with evident cleavage planes in the cells. Sometimes the sliding plane due to folding of cells is about 45°–65° is from the transversal side, sometimes there is a buckling on the end-grain section. In the most severe cracks conditions there is a wood tissue divarication (b2) and looking to the cross-cut section there is a mixed dislocation between the transversal and radial surface (b4). The rupture occurs at the end of the ring width in the transition from the last cells of the latewood to the vessels of earlywood formed the following year (arrows in b5). In the most severe cracks there is a divarication among the tissues. Following the cracks, the starting point was evident in the cell walls of the largest vessels in latewood, which become the weak point of chestnut tissue (c3). The rings of such samples show a less abrupt transition from the vessels of earlywood to the vessels of latewood which show a quite remarkable size. The cracks spread in the rupture of the cell wall in the vessel in latewood following mainly the parenchymatic ray pattern.

### FT-IR

**Figure 5:**
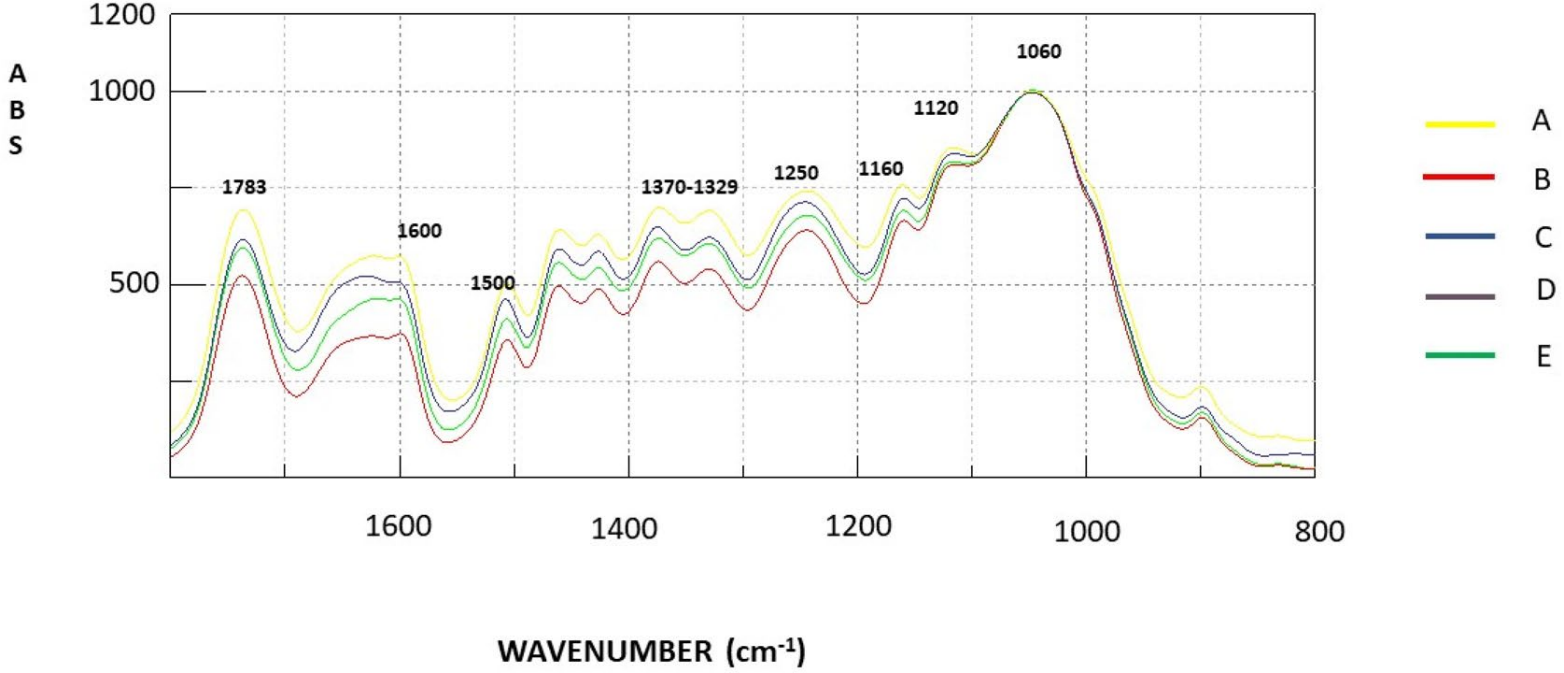
FTIR normalized spectra of A–E areas of chestnut coppices of Amiata mountain of 11–14 years old.

From Fig. 5, the IR spectra can be observed of chestnut representative samples that feature similar profiles among all investigated stands, which means no relevant, different chemical composition was detected in the different stands; some differences are evident in the intensity of the signals in some regions of the spectrum exclusively from site A. Noteworthy the peak at 1200 cm^−1^ is related to syringyl ring which is typic of hardwoods. The peak wave numbers as reported in Pandey^37^ to be related to the lignin and cellulose IR bands are summarized in Table 4. Semi-quantitative analysis based on the peak values suggest some differences in the ratio holocellulose/lignin.

**Table 4:** Peak height and area of five different chestnut stands on the Amiata mountain according to Pandey (1999) and Naumann et al. (2007)^47^.

Peak wave number (cm^−1^)	A		B		C		D		E	
	Height	Area	Height	Area	Height	Area	Height	Area	Height	Area
1030	0.213	11.830	0.240	11.830	0.219	12.962	0.237	13.110	0.215	12.385
1120	0.0795	2.287	0.081	2.256	0.075	2.060	0.071	1.987	0.072	2.041
1160	0.102	2.333	0.103	2.151	0.099	2.418	0.094	2.045	0.104	2.399
1200	0.161	9.587	0.175	9.465	0.196	10.875	0.158	8.847	0.188	10.657
1329	0.075	2.038	0.071	2.083	0.067	1.929	0.072	2.041	0.071	1.940
1370	0.091	2.334	2.396	0.081	0.106	2.559	0.092	2.430	0.1040	2.423
1740	0.226	12.900	0.281	16.488	0.208	11.556	0.264	12.823	0.232	11.003
1500	0.085	2.348	0.093	2.330	0.072	2.077	0.114	3.251	0.088	2.847
1600	0.155	9.086	0.19	12.531	0.138	9.353	0.116	10.830	0.12	6.81
1740/1500	2.65	5.49	3.02	7.07	2.88	5.56	2.31	3.94	2.63	3.86
1740/1600	1.45	1.41	1.47	1.31	1.50	1.23	2.27	1.18	1.93	1.61
1740/(1500 + 1600)	0.94	1.12	0.99	1.10	0.99	1.01	1.14	0.91	1.11	3.85
1500/1600	0.54	0.25	0.48	0.18	0.52	0.22	0.98	0.30	0.73	0.41

1740 cm^−1^ = Holocellulose band, 1500 cm^−1^ = Band related to wood lignin content, 1600 cm^−1^ = contributions from conjugated C–O group; 1329 cm^−1^ C–H vibration in cellulose and C–O syringyl derivates, 1371 cm^−1^ CH deformation in cellulose and hemicellulose; 1200 cm^−1^ syringyl ring and C–O stretch in lignin and xylan, 1160 cm^−1^ C–O–C vibration in cellulose and hemicellulose, 1320 cm^−1^ C–O vibration in cellulose and hemicellulose.

Higher wood holocellulose (cellulose and hemicellulose) from site B is indicated by a strong carbonyl band at 1740 cm^−1^. Stand E is characterized by the highest lignin content, estimated from the relative peak height and area of 1505 cm^−1^, known as the lignin characteristic peak. The holocellulose-to-lignin ratio (1740/1500) reveals B with a prevalence of first value with respect to lignin, while in D, there is the opposite condition. The differences are not significant by the statistical point of view.

## Discussions

The analysis of wood physical and mechanical properties in chestnut coppices of Monte Amiata has provided values comparable with those reported in other Italian provenances^2, 23,25^. The values also find agreement with^4^, who refer to chestnut wood of young trees (ρ_12_ = 556 kg/m^3^); the D stand has density values that are comparable to more adult trees at 31-years old at the site of Castelli Romani as reported in^2^, that is a very positive result because it means it is possible to compare the physical and mechanical properties of young shoots with the more adult trees.

The selected stands differ from each other significantly in terms of certain dendrometric parameters, specifically the number of stools per hectare, the number of shoots per stool, shoot diameter and shoots height^32^. As evidenced by the PCA analysis, the stands do not group so much each other in the investigated wood physical and mechanical properties, suggesting that the individual (plant) behaviour prevails over the stand characteristics.

Neverthless wood density by ANOVA proves to be significantly different among some of the stands and a negative correlation was established between wood density and dominant height and diametric growth this last can be associated to the ring width parameter (TRW). Noteworthy in recent times also in other ring-porous species, like *Quercus*, the well-known positive correlation between tree ring width (i.e., growth) and density^38^ has evidenced to be reconsidered because it has been shown to be poor or even absent^39^. According to the obtained results, it seems possible to indicate stand density, here represented by the number of stools/ha, as a factor affecting moderately and positively wood density. Few references deal directly with the effect of forest stand density on physical and mechanical wood properties, and the few results available in the references do not seem comparable at all with our study. In planted Eucalyptus^20^, for example, a higher tree spacing has shown to increase in wood density, exactly the opposite of what we obtained in our study. Noteworthy the obtained result is consistent with previous research on chestnut^2^, which shows that higher competition negatively influences TRW (i.e., diameter and basal area), and there is also a negative correlation among TRW and wood density. The result obtained with the investigated chestnut coppices can support forest management decisions pertaining to the investigated wood technological characteristics. Lower competition expressed as the number of stools/ha and higher tree height decreases the value of wood density and subsequent mechanical properties. The example is in the D stand which is characterized by low values in the height tree curves, and for the opposite, the highest ρ_12_ and MOR. As wood density is indicated as a parameter that can increase the strength to the climatic stress^8^, we would have to assume a higher competition means greater probability of resilience. Nevertheless, the explanation seems too simplistic because other mechanisms, namely water use efficiency or transpiration might indicate better the impact of climatic changes on the species resilience^32^. The results boost new researches in possible correlations among ecophysiological indicator and wood technological parameters to assess the strength to the stressing environmental conditions.

Wood density has been demonstrated to be the most important variable connected to stand characteristics, while the other examined physical (shrinkages) and mechanical properties (MOR and compression strength) are posed at a secondary level and their role is determined mainly by the significant positive correlation with ρ_12_ values. The positive and significant correlation between wood density and shrinkages as well as mechanical properties find full agreement with other similar studies on chestnut from different provenances^2,6,25,40^, and in general reflect the state of the art for the most ring-porous species^38^. In this case, the MOR value seems less related to ρ_12_ and this in an agreement with the study of^41^. The study is also a step towards a link between phenotypic characters and wood physical and mechanical properties^9,10^.

FT-IR techniques, including traditional infrared spectroscopy (FT-IR), has been used as a routine method for obtaining rapid information on the structure of wood constituents^42^. FTIR has been used more times to characterize the chemistry of wood, however most published work describes softwood species FT-IR spectra, and very little information is available regarding hardwood, particularly chestnut. This study marks the first time that FT-IR was applied to investigate wood quality in chestnut, though FTIR has not provided a difference in the quality of the peaks, testifying to a common chemical matrix among stands. As the stands are in the same geographical area and the soil has a similar chemical matrix, we had to expect this specific result because soil has supposed to play a fundamental role in wood chemical composition in chestnut wood^24^. Certain differences are found among the semi-quantitative values, reflecting the ratio of cellulose to lignin. In this case, we are dealing with the amount of components in the cell walls that can be different owing to the presence of tension wood and a different cell wall formation and final cell wall thickness^28,43, 44^. FTIR spectroscopy associated with wood density measurements has proven to be a promising alternative to traditional methods for screening of individual- or species-specific resistance to embolism in angiosperms^30^. For this reason, further research is needed to fully explore the potential of the technique in this field.

## Conclusion

In chestnut stands, physical and mechanical properties seem more related to individual behaviour than to stand features. A moderately positive correlation between the number of stools per hectare and wood density, along with a negative correlation of shoot height and diameter with density, has been assessed in the investigated stands. Based on these assumptions, it is possible initiate new management perspectives considering the effect of the number of stools/ha and shoot/height as the most promising characteristics related to the wood characteristics and the possible effect on the resilience of chestnut coppice stand based on wood physical and mechanical measurements. We also have to take into account that in the investigated samples, juvenile wood is present and this makes the research even more interesting owing to the chance to study and use young shoots in chestnut coppice.

## Material and methods

### Study site

The study was carried out in some publicly owned young chestnut coppices (10–15 years) on Monte Amiata, Siena (Central Italy) growing in a range of altitude between 990 and 1145 m a.s.l. on volcanic soil. Monte Amiata (Siena) is an important area for chestnut cultivation; the species grows here under ecologically suitable conditions^45^. Climate data for the period 2004–2016 from 'Le Vigne' meteorological station (450 m a.s.l.; 723370 E UTM, 4744226 N UTM) show an annual rainfall of 1117 mm and annual average temperature of 13.2 °C. Vegetation is characterized by chestnut dominance. Traditional forest management involves coppicing with standards with a rotation age of 20–22 years, and a thinning from below between 12- and 15-years old, only under public ownership.

### Field sampling

The main dendrometric parameters were assessed in five circular sampling areas, with a ray of 15 m, located in Cipriana (sample area C), Sant’Antonio (A and B), Le Decine (D) and Acquagialla (E). Diameter at breast height of all trees and a sample of 15 tree heights representative of the diametric classes were measured in each sample plot.

For the suppressed and intermediate stand layers, 15 shoots with diameter at breast heigth (dbh) > 10 cm were selected and harvested in each plot. The materials were used for the characterization of chestnut wood deriving from small and medium size trees that are ordinarily eliminated by thinning. The samples were above the curvature area in order to avoid tension wood.

### Physical and mechanical properties

From each thinned shoot, sample logs of 40 cm in length were taken and one board was cut according to a radial pathway. The samples were then conditioned at the temperature of 20 ± 2 °C and at 65 ± 5% relative humidity until the boards reached an equilibrium moisture content of roughly 12%. One sample in each board containing approximately rings from 4 or 5 to 10 cm from the pith was cut with a size of 20 × 20 × 300 mm. The sample was used to measure bending strength (MOR) according to UNI ISO 3133. Furthermore, two cubic samples of size 20 × 20 × 30 mm were sampled in order to measure density at 12% (ρ_12_) and compression strength (σ12) according to UNI ISO 3787 and total shrinkage (βV). By the original board, a sample of 50 × 50x20 mm according to UNI EN 1534 was cut in order to measure hardness by the Brinell method. For this last test, a 10-mm diameter carbide ball was pressed with a force of 1000 N into the test sample for 15 s, and the load was applied to the tangential face and at the end, the diameter of the footprint was measured.

### FT-IR analysis

Before FT-IR analysis, chestnut wood samples, randomly selected, were ground in a cutting mill (IKA MF 10.1, IKA Were GmbH & Co. KG, Staufen, Germany) to pass through a 0.5 mm sieve and then oven-dried for three hours at 60 °C (FD 115, Binder GmbH, Tuttlingen, Germany). Potassium bromide (KBr) pellets (diameter 13 mm) were prepared with a sample concentration of 2% using a Specac mini-pellets press at 2 bar for 5 min (Specac Inc., Fort Washington, USA). FT-IR spectra were recorded in absorption mode in the range of 4000–400 cm^−1^ with resolution of 4 cm^−1^ using a FT-IR-4100 FT-IR spectrometer (Jasco Corporation, MD, USA).

All FT-IR spectra were baseline-corrected and normalized to the most intense peak of the spectra (1053 cm^−1^) using Jasco Spectra Manager software (v2.14.02). Peak height and area were measured with the same software after constructing a baseline connecting the lowest data points on either side of the respective peak^46^.

### Statistical analysis

Statistical analyses were carried out with Past p3 (PAleontological STatistical software, version 3.09, University of Oslo, Norway) in order to determine the effect of dendrometric parameters on physical and mechanical properties.

The differences among the five studied areas from the dendrometric perspective were subjected to analysis of variance (ANOVA) and principal component analysis (PCA). The former was carried out to determine whether the five areas were significantly different in their dendrometric values.
